# Analgesic-like Activity of Essential Oils Constituents

**DOI:** 10.3390/molecules16032233

**Published:** 2011-03-07

**Authors:** Damião Pergentino de Sousa

**Affiliations:** Department of Physiology, Federal University of Sergipe, CEP 49100-000, São Cristóvão, SE, Brazil; E-Mail: damiao_desousa@yahoo.com.br

**Keywords:** analgesic, antinociceptive, essential oils, terpenes, natural products

## Abstract

Research on neuroactive drugs is a pharmaceutical sector of high interest and growth. The discovery of efficient drugs that can relieve pain is a subject of research in the pharmaceutical industry and academic field because pain is a symptom of many diseases. This review will summarize results on the discovery of essential oil constituents with analgesic-like activity from the chemical and pharmacological perspectives. Overall, 43 bioactive compounds were selected in nociception models. Among them, 62.8% were monoterpenes, 18.6% sesquiterpenes and other constituents represented 18.6%. The data show the potential of this group of natural product chemicals as analgesic drugs that may be useful for therapeutic purposes.

## 1. Introduction

Pain is defined as an unpleasant sensory and emotional experience associated with actual or potential tissue damage, or described in terms of such damage [1]. It is a sensation described as a multidimensional experience, in which several components are involved: motivational, emotional, sensory-discriminative, affective, and cognitive aspects [2]. Perception of pain and response to analgesic drugs are complex processes that involve multiple biochemical pathways. Each of these pathways is influenced by significant genetic factors that may modify pain perception and/or response to analgesics. Indeed, there is a wide range of interindividual variability in the perception of pain, as well as in the dosage of analgesics that will provide pain relief [3].

A significant number of the world population is affected by some kind of pain, causing loss of good quality of life. Developing of treatments for pain relief has been the motivating factor behind many studies carried out by academic investigators and by the pharmaceutical industry in response to the demand for powerful analgesics and that exhibit their pharmacological response through new mechanisms of action and with less side effects. In a review article 202 species of plants with analgesic activity involving 79 botanic families were reported. Most studies were conducted using rats and/or mice in experimental models of pain using extracts from plants [4]. This information shows the potential of plants as sources of drugs with analgesic effects. For thousands of years medicine and natural products have been closely linked prominently through the use of traditional medicines [5,6,7]. In fact, clinical, pharmacological, and chemical research of these traditional medicines, which are derived predominantly from plants were the basis of many drugs [5,6,7,8]. Medicinal plants contain a diversity of biologically active compounds that belong to different natural product chemical classes such as terpenes, saponinic glycosides, steroids, alkaloids, and flavonoids [5,9,10]. 

**Figure 1:**
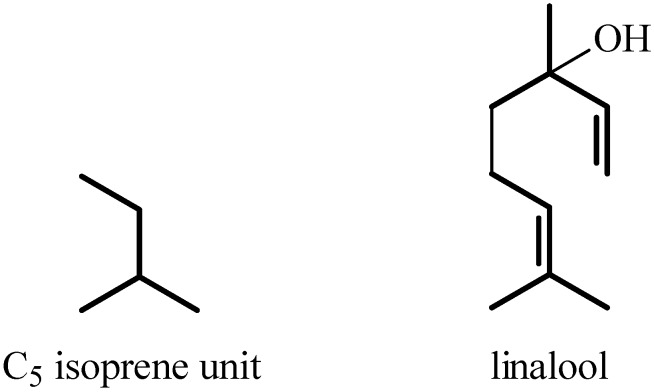
Chemical structures of the isoprene unit and linalool.

The antinociceptive profiles of many plant species widely used in folk medicine, such as *Hyptis pectinata* Poit. (Lamiaceae) [11], *Hyptis fruticosa* Salzm. ex Benth. (Lamiaceae) [12], and *Erythrina velutina* Willd. (Fabaceae) [13] have been studied. Many of these species are aromatic plants and contain bioactive essential oils. These oils are natural products composed of volatile and lipophilic compounds. They are often obtained by heating the leaves, petals or other plant parts in steam and collected as an oily fraction. In general, essential oils consist of chemical mixtures involving several tens to hundreds of different types of molecules. Only a few have a single component in high percentage. The constituents of essential oils are compounds that have low molecular weight and are used to give flavor in foods and as fragrances in the cosmetics industry. Chemically, the oils are composed mainly of terpenoids and phenylpropanoids, including polyketides and very few alkaloids. The terpenoids form a large family derived from C_5_ isoprene units, such as linalool which consists of two isoprene units (Figure 1). They are classified depending on the number of isoprene units as hemiterpenes, monoterpenes, sesquiterpenes, diterpenes, sesterterpenes, triterpenes and tetraterpenes. They are formed by two biosynthetic pathways, by way of the intermediates mevalonic acid or 1-deoxy-D-xylulose 5-phosphate. The terpenoids from essential oils are often monoterpenes and sesquiterpenes, but the most abundant terpenoids in essential oils are monoterpenes, which account for about 90% of the oils. Diterpenes can be present when the essential oils are extracted with organic solvents. The phenylpropanoids and derivatives are formed from shikimic acid [5,14]. Compounds derived from these chemical classes or who belong to other chemical groups may be present in oils often in lower amounts.

Many essential oils are found to exhibit varied biological properties [14], such as spasmolytic [15], anxiolytic [16], anticonvulsant [17] and antinociceptive [18] activity. These effects are probably due to high structural diversity of the essential oil constituents. The study of each individual chemical component is critical to understand its mechanism of pharmacological action and toxicity, including potentially beneficial clinical effects on human health. Considering that the essential oils and their constituents are common in many plant species and are used in cosmetic and pharmaceutical preparations, as well as in the food industry, it is important to review the pharmacological potential of the essential oils constituents with analgesic-like activity.

## 2. Methodology

The present study was carried out based on the literature review of the compounds from essential oils with analgesic-like activity. Information about 43 bioactive compounds is given in Table 1. These components must contribute to the analgesic-like activity of essential oils and plant extracts in which they are found. This list is organized by chemical groups mentioning the experimental model, administration route, animal tested and dose used, as described in the literature. Chemical structure and name of bioactive compounds, as well as the corresponding references are provided. 

The compounds presented in this review were selected based on the effects shown in specific animal models for evaluation of the antinociceptive activity and/or by complementary studies, which have aimed to elucidate the mechanisms of action. To select the essential oils constituents terms related to the theme, such as “essential oils”, “monoterpenes” and “phenylpropanoids” were used, as well as names of representative compounds of these chemical groups refining with “analgesic” or “antinociceptive”. A search was performed in the scientific literature databases Chemical Abstracts and PubMed until December 2010. The antinociceptive activity was accepted when the essential oil constituents had shown effects in different pain models, including the writhing, formalin, tail-flick, tail immersion, and hot plate model. 

## 3. Results and Discussion

Among the 43 compounds selected, 62.8% were monoterpenes, 18.6% sesquiterpenes and other constituents represent 18.6%. The routes of administration of the constituents tested were intraperitoneal (i.p.), subcutaneous (s.c.), oral (p.o.), intraplantarly (i.pl.), intracerebroventricularly (i.c.v.), intrathecal (i.t.), and intragastrically (i.g.). 

Considering the difficulty of isolating the essential oils constituents in sufficient quantity and high purity to study using *in vivo* animal models, most of the compounds tested were purchased from chemical companies. In fact, in recent years there was a significant increase at antinociceptive studies of this chemical group. The commercial constituents from chemical companies are isolated from the plants that biosynthesized them as major components. The constituents found in essential oils at small quantities are synthesized on a large scale using as raw materials the secondary metabolites abundant in Nature. The commercial availability of natural compounds has allowed the extensive pharmacological study and proposed antinociceptive mechanism of action of some essential oil constituents. The studies with pure compounds usually do not present the difficulties encountered in the evaluation of essential oils, such as large variability in the chemical composition or presence of toxic components.

The terpenes and others chemical groups found at essential oils are structurally simple molecules, but reported studies of their pharmacological proprieties show their potential clinical use as analgesic drugs. All are low molecular weight compounds, usually with high lipid solubility. They can penetrate the blood-brain barrier and act in the central nervous system. Therefore they may present a profile of psychoactive drugs. It is possible that other essential oil constituents that have not been evaluated in experimental models must have pharmacological activity due to its structural similarity to the bioactive constituents. Terpenes not found in essential oils, such as sesterterpenes and sesquiterpenes, but with similar chemical characteristics, are potentially bioactive in pain models. Synthetic compounds prepared from monoterpenes also exhibit analgesic-like properties. The pharmacological potency has been higher in relation to their natural precursors [19,20,21,22].

Studies of proposed analgesic-like activity mechanisms have been conducted with some essential oil constituents that are widely known because of their uses in the pharmaceutical, cosmetic, and food industries. For example, (-)-linalool, a major component of many plant essential oils, such as *Aniba rosaeodora* (Lauraceae) [23], acts on several receptors, including opioids, adenosine A1 and A2, cholinergic M2, and produces changes in K^+^ channels [24,25,26,27]. Experiments at animal models have shown that the analgesia of (-)-menthol, a compound found in *Mentha* species such as *M. piperita* and *M. arvensis*, occurs via an opioid receptor [8]. Studies also showed the topical analgesic effects of (-)-menthol [28]. In fact, pharmaceutical preparations containing menthol are used for topical pain relief. When menthol is applied to the skin or mouth at low concentrations, it elicits a pleasant cool sensation. Eugenol, a phenylpropanoid, is also a topical analgesic agent widely used in the dental clinic. It is the analgesic principle from *Abutilon indicum* [29]. Studies suggest that eugenol may exert their analgesic effects via the capsaicin receptor and high-voltage-activated calcium channel inhibition [30,31]. 

The structure-activity relationships of some compounds have been established. For example, it was shown that the absence of the ketone or epoxide groups on the structure of the monoterpene rotundifolone, an important chemical constituent of the essential oil of the plant *Mentha* x *villosa* Hudson (Lamiaceae), known popularly as “hortelã-da-folha-miúda”, did not decrease the antinociceptive activity. Indeed, there is a significant increase of this pharmacological activity. In this study, De Sousa [32] showed that both functional groups contribute to the antinociceptive activity of rotundifolone and that the presence of the epoxide or ketone groups in the structure of rotundifolone is not a critical requirement, but rather the position of these groups on the molecule affects its antinociceptive activity. The study was conducted by comparing the antinociceptive activity of structurally similar monoterpenes. Differences in pharmacological potency also have been observed between monoterpene stereoisomers. For example, the (-)-3-isothujone was approximately fifteen times more potent than (+)-3-thujone, while the antinociceptive action of racemic 3-isothujone was less effective than that of the (-)-3-isothujone enantiomer. 3-Thujone and (-)-3-isothujone are ketonic constituents of the essential oils of two *Artemisia* species, *A. absinthium* L. and *A. pontica* L. (Compositae) [33]. Pharmacological assessment of chiral compounds in an early research phase can lead to the selection of a single isomer for development. This selection process can maximize the potential for specific activity and minimize the potential for side-effects. The enantiomers of menthol are another example of the influence of stereochemistry on the pharmacological action of monoterpenes. Contrary to what was observed for (-)-menthol, its enantiomer, (+)-menthol, was unable to modify the pain threshold in hot-plate and writhing tests [8]. However, it is important to consider that the inactivity or lower pharmacological potency observed for stereoisomers could vary widely according to the experimental model, animal, route of administration, and dose used [33,34]. 

Among the main models of pain used in the pharmacological evaluation of the constituents, there is the formalin test that is a model of acute and tonic pain, being considered a more valid model for clinical pain than tests involving mechanical or thermal stimulation [35]. The first phase results from direct chemical stimulation of the nociceptive afferent fibers, mainly C fibers, and the release of substance P [36] that may be inhibited by centrally acting analgesics such as morphine. The second phase results from the action of inflammatory mediators released locally, such as prostaglandins, serotonin, histamine, and bradykinin [37,38] and also from enhanced synaptic transmission in the spinal cord [39]. Drugs whose principal mode of action is central, inhibit both phases of this test, whereas peripherally acting drugs only inhibit the second phase. Another pain model is the tail flick test, whose response may be a spinally mediated reflex [40]. It is a very sensitive test to drugs acting on central nervous system. Moreover, it was shown that the effectiveness of analgesic agents in the tail flick pain model is highly correlated with relief of human pain [41]. Meanwhile the hot plate test is a specific central antinociceptive test with response of cerebral cortex or spinal cord integration [42]. Another test, the writhing test, it is a pain model widely used in the screening of analgesic drugs, however it has low specificity. Other classes of drugs, such as anti-inflammatory and non-steroidal antihistamines also inhibit the contortions behavior [43,44]. In the tail immersion test the central analgesic drugs are effective in increasing the reaction time for tail removal of the animals, which suggests an antinociceptive action [45,46]. 

Several studies show that compounds found in essential oils are quickly absorbed after dermal, oral, or pulmonary administration. The majority are metabolized and eliminated by the kidneys in the form of phase-II conjugates. A small fraction is eliminated unchanged by the lungs. For example, after oral administration of (-)-menthol, 35% of the original menthol content was excreted renally as menthol glucuronide. Some pharmacokinetic studies show that these compounds also are quickly eliminated after intravenous application, with an elimination half life of about one hour [47].

The occurrence of the toxicity of some essential oils seems related to presence of monoterpene ketones such as camphor and pinocamphone. Clinical intoxications induced by essential oils were characterized by tonico-clonic or solely clonic convulsions [48]. There are reports of such symptoms in adults and children who used essential oils for therapeutic purposes [49]. The presence of one of these compounds or another chemically related as major components of any essential oil may to cause human health risks. Therefore, toxicological studies should be applied to essential oils and their components that are used for therapeutic purposes. 

**Table 1:** Essential oil constituents with analgesic-like activity.

Compound	Experimental model/ administration route	Animal tested			Dose or conc. used or ED_50_ [ref.]
**Monoterpenes**					
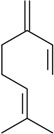	Hot plate test/i.p. or s.c.	Mouse			10–40 mg/kg [50]
	Writhing test/i.p. or s.c.				
	Rat paw hyperalgesic test/p.o.	Rat			5–135 mg/kg
	Writhing test/p.o.	Mouse			15–405 mg/kg [51]
*β*-Myrcene					
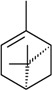	*p*-Benzoquinone-induced abdominal	Mouse			500 mg/kg [52]
	constriction test/p.o.				
	Tail-flick test/i.p.	Mouse			0.05–0.2 mL/kg [53]
*α*-Pinene	Formalin test/p.o.	Mouse			400 mg/kg [54]
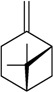	Tail-flick/i.p.	Mouse Rat			0.3 mg/kg [55]
	Hot plate test/i.p.				
*β*-Pinene					
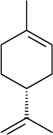	Writhing test/i.p.	Mouse			25–50 mg/kg [56]
	Formalin test/i.p.				
	Writhing test/i.p.	Mouse			250 mg/kg [32]
(+)-Limonene					
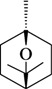	Writhing test/p.o.	Mouse			100–400 mg/kg [57]
	Formalin test/p.o.				
	Tail-flick/i.p.	Mouse Rat			0.2–0.5 mg/kg, [55]
1,8-Cineole	Hot plate test/i.p.				
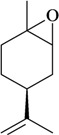	Writhing test/i.p.	Mouse			250 mg/kg [32]
Limonene oxide					
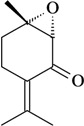	Writhing test/i.p.	Mouse			125–250 mg/kg [58]
	Tail-flick test/i.p.	Rat			
	Tail-immersion test/i.p.				
	Writhing test/i.p.	Mouse			250 mg/kg [32]
	Writhing test/p.o.	Mouse			10–200 mg/kg [59]
Rotundifolone	Formalin test/p.o.				
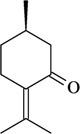	Writhing test/i.p.	Mouse			250 mg/kg [32]
(+)-Pulegone					
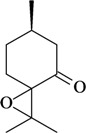	Writhing test/i.p.	Mouse			250 mg/kg [32]
Pulegone oxide					
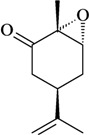	Writhing test/i.p.	Mouse			250 mg/kg [32]
Carvone epoxide					
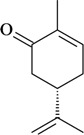	Writhing test/i.p.	Mouse			250 mg/kg [32]
(+)-Carvone					
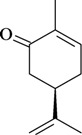	Writhing test/i.p.	Mouse			50–200 mg/kg [34]
	Formalin test/i.p.				
	Writhing test/i.p.	Mouse			250 mg/kg [32]
(-)-Carvone					
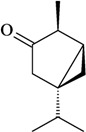	Hot plate test/s.c.	Mouse			ED_50_ = 100 mg/kg [33]
	Nilsen test/s.c.				
(+)-3-Thujone					
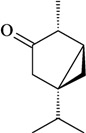	Hot plate test/s.c.	Mouse			ED_50_ = 6.5 and 14.1 mg/kg [33]
	Nilsen test/s.c.				
(-)-3-Isothujone					
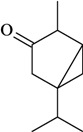	Hot plate test/s.c.	Mouse			ED_50_ = 16.7 mg/kg [33]
(±)-3-Isothujone					
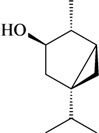	Hot plate test/s.c.	Mouse			ED_50_ = 33.3 mg/kg [33]
(-)-3-Isothujanol					
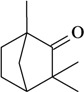	Tail-flick test/i.p.	Mouse			0.05–0.2 mL/kg [52]
Fenchone					
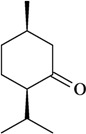	Writhing test/-	Mouse			---------- [60]
(+)-Menthone					
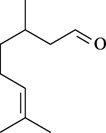	Writhing test/i.p.	Mouse		50–200 mg/kg [61]	
	Formalin test/i.p.				
	Hot plate test/i.p.				
	Formalin test/i.p.	Mouse		50–200 mg/kg [62]	
	Capsaicin-induced nociception test /i.p.				
(±)-Citronellal	Glutamate-induced nociception test/i.p.				
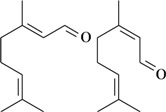	Formalin test/p.o.	Rat		30–1000 mg/kg [63]	
(geranial) (neral)					
Citral (= geranial + neral)					
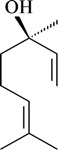	Writhing test/s.c.	Mouse		25–100 mg/kg [24]	
	Hot plate test/s.c.				
	Formalin test/ s.c.	Rat Mouse		50–150 mg/kg [27]	
	Hot plate test/s.c.				
	Glutamate-induced nociception test/i.p. or p.o. or i.t. or i.pl.	Mouse		5–200 mg/kg, 0.1–3µg/site, 10–300 ng/paw; 200 mg/kg [64]	
(-)-Linalool	Biting response induced by glutamate, AMPA, NMDA, kainate and substance P tests/i.p.				
	CFA-induced persistent inflammation/i.p.	Mouse		50–200 mg/kg [65]	
	Partial sciatic nerve ligation–induced neuropathic Hypersensitivity/i.p.				
	Nociception induced by pro-inflammatory cytokines/i.p.				
	Paw withdrawal test/s.c.	Rat		50–200 mg/kg [66]	
	Carrageenan- or L-glutamate- or prostaglandin E_2_-evoked thermal hyperalgesia				
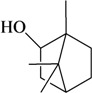	Hot plate/-	-		ED_50_ = 488.14 mmol/L [67]	
Borneol					
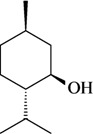	Hot plate test/p.o.	Mouse		1–10 mg/kg;	
				5–10 µg per mouse [8]	
(-)-Menthol	Writhing test/p.o. or i.c.v.				
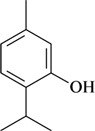	Hot plate test/p.o	Mouse		1–100 mg/kg [68]	
Thymol					
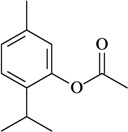	Hot plate test/p.o	Mouse		0.31–17.7 mg/kg [68]	
Thymyl acetate					
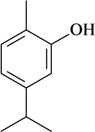	Writhing test/i.p.	Mouse		25–100 mg/kg [69]	
	Formalin test/i.p.				
	Hot plate test/i.p.				
	Glutamate-induced nociception test/i.p.				
Carvacrol	Capsaicin-induced nociception test/i.p.				
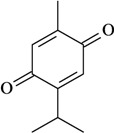	Formalin test/i.p. or p.o. or i.c.v.	Mouse		1–10 mg/kg or 1–4 µg/mouse [70]	
Thymoquinone					
**Sesquiterpenes**					
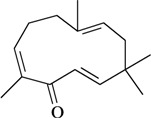	Writhing test/i.p.	Mouse		10–100 mg/kg [71]	
Zerumbone	Hot plate test/i.p.				
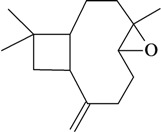	Writhing test/i.p.	Mouse		12.5–25 mg/kg [72]	
	Hot plate test/i.p.				
Caryophyllene oxide					
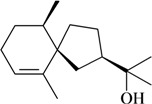	Writhing test/i.p.	Mouse		50 mg/kg [73]	
Agarospirol					
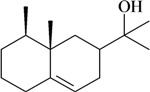	Writhing test/i.p.	Mouse		50 mg/kg [73]	
Jinkoheremol					
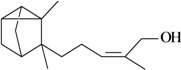	Writhing test/i.p.	Mouse		50 mg/kg [73]	
*α*-Santalol					
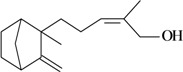	Writhing test/i.p.	Mouse		50 mg/kg [73]	
*β*-Santalol					
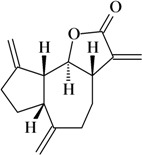	Writhing test/i.p.	Mouse		50 mg/kg [73]	
Dehydrocostus lactone					
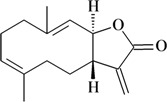	Writhing test/i.p.	Mouse		50 mg/kg [73]	
Costunolide					
**Phenylpropanoids and other chemical constituents**					
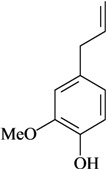	Writhing test/i.p.	Mouse	1–100 mg/kg [74]		
	Formalin test/i.p.				
	Hot plate test/i.p.				
	Writhing test/p.o.	Mouse	10–50 mg/kg [29]		
	Tail-flick/p.o.				
	Formalin test/i.t.	Mouse	12.5–50 µg/2.5 µL [30]		
	Writhing test/i.t.				
Eugenol	Writhing test/p.o.	Mouse	50–100 mg/kg [75]		
	Hot plate test/p.o.				
	Writhing test/s.c.	Mouse	50 mg/kg [76]		
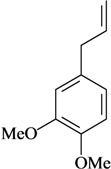	Formalin test/p.o.	Mouse	3–10 mg/kg [77]		
Methyleugenol					
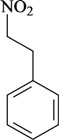	Writhing test/i.p.	Mouse	15–50 mg/kg [78]		
1-Nitro-2-phenylethane	Formalin test/i.p.				
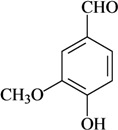	Writhing test/p.o.	Mouse	1–10 mg/kg [79]		
Vanillin					
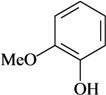	Formalin test/i.t.	Mouse	25–150 µg/2.5 µL [30]		
Guaiacol	Writhing test/i.t.				
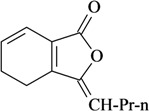	Writhing test/i.g.	Mouse	2.5–10 mg/kg [80]		
Ligustilide	Formalin test/i.g.				
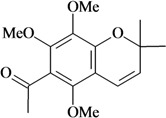	Writhing test/p.o.	Mouse	50–100 mg/kg [81]		
Evodione	Tail immersion test/p.o.				
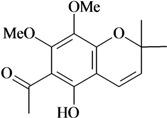	Writhing test/p.o.	Mouse	50–100 mg/kg [81]		
Leptonol	Tail immersion test/p.o.				

In Table 1 there are bioactive molecules such as *β*-myrcene, *α*- and *β*-pinene, and (+)-limonene that only contain C and H atoms in their chemical structures. These hydrocarbons have shown pharmacological activity in various pain models and by different routes of administration. Interestingly, the presence of a heteroatom on the chemical structure of monoterpenes is not a critical requirement to have antinociceptive activity. Table 1 presents the structures of molecules containing different chemical functional groups, for example, ketones, aldehydes, alcohols, ethers, quinones, lactones, and aromatics. Among mono-oxygenated compounds there are the ethers 1,8-cineole, limonene oxide, and caryophyllene oxide; the alcohols (-)-linalool, (-)-menthol, (-)-3-isothujanol, borneol, *α*- and *β*-santalol, jinkoheremol, and agarospirol. *α*-Santalol is a sesquiterpenoid isolated from Saussurea root (*Santelum album* L.). It is an “Oriental incense” used for meditation and sedation. It was proposed that *α*-santalol might cause analgesia in mice because of an inhibitory binding effect on the δ_2_-opioid receptor [73]. Among the carbonyl compounds, there are the monoterpenes (-)- and (+)-carvone, fenchone, (+)-menthone, (+)-pulegone, (+)-3-thujone, (-)- and (±)-3-isothujone, zerumbone, citral, and (±)-citronellal. Zerumbone is the main component of *Zingiber zerumbet* Smith. It was suggested that the opioid system is involved in its antinociceptive mechanism of action [71]. In Table 1 there are also the phenols thymol and carvacrol. These isomeric monoterpenes are major components found in essential oils of various species of genus *Thymus* [82], such as *Thymus vulgaris*, whose essential oil has antinociceptive activity [83]. The di-oxygenated compounds are rotundifolone, pulegone oxide, carvone epoxide, thymyl acetate, dehydrocostus lactone, costunolide, eugenol, methyleugenol, guiacol, thymoquinone, and ligustilide, one of the main compounds of Danggui (*Angelica sinensis* (Oliv.) Diels) essential oil. This plant is one of the most important traditional Chinese herbs, has been widely used in prescriptions in Traditional Chinese Medicine [80]. Thymoquinone is the major component of *Nigella sativa* oil. It was suggested that this oil and thymoquinone produce antinociceptive effects through indirect activation of the supraspinal µ_1_- and k-opioid receptor subtypes [70]. Vanillin is the only tri-oxygenated essential oil constituent with reported antinociceptive activity. The penta-oxygenated compounds are two chromenes that were isolated from *Melicope lunu-ankenda* leaf oil as major constituents, evodione (20.2%) and leptonol (22.5%) [81]. Although it is rare to find nitro derivatives in higher plants, 1-nitro-2-phenylethane was the main component (71%) of the essential oil of *Aniba canelilla*. Preliminary studies showed that the essential oil of this plant has antinociceptive activity in mice. It was suggested that the pharmacological activity of 1-nitro-2-phenylethane in pain models is probably of peripheral origin [78]. 

Table 1 also shows that many compounds such as *β*-pinene, 1,8-cineole, and thymoquinone are bioactive at low doses [55,70], while (-)-3-isothujone presented an ED_50_ = 6.5 [33]. A comparative analysis of the constituents cannot however be done due to the different experimental conditions and variability of methods and routes of administration used in the evaluation of these compounds. Nevertheless, the data show that most chemical classes of compounds found in essential oils are effective in pain models. They must act by different mechanisms of action that merit further investigation. The future outlook for the development of new analgesic drugs derived from essential oils is therefore positive.

## 4. Conclusions

Considering that the relief of painful symptoms typically involves pharmacotherapy and there are hundreds of constituents of essential oils that have not been assessed, these continue to be a major source of bioactive compounds in pain models. The chemical diversity of this group of natural compounds, as well as the possibility of exhibiting antinociceptive activity via different mechanisms of action should stimulate interest in studying them through a chemical and pharmacological approach aimed at clinical use, as already happens with some of the compounds discussed in this review. The data presented in this review should also prove useful as the basis for the study of structurally similar compounds with improved pharmacological profiles.

## References

[1] IASP Pain Terminology http://www.iasp-pain.org/AM/Template.cfm?Section=Pain_Definitions&Template=/CM/HTMLDisplay.cfm&ContentID=1728#Pain.

[2] Mersky Y.H. (1986). Classification of chronic pain. Descriptions of chronic pain syndromes and definitions of pain terms. Prepared by the International Association for the Study of Pain, Subcommittee on Taxonomy. Pain Suppl..

[3] Shi Q., Cleeland C.S., Klepstad P., Miaskowski C., Pedersen N.L. (2010). Biological pathways and genetic variables involved in pain. Qual. Life Res..

[4] Almeida R.N., Navarro D.S., Barbosa-Filho J.M. (2001). Plants with central analgesic activity. Phytomedicine.

[5] Dewick P.M. (2001). Medicinal Natural Products: A Biosynthetic Approach.

[6] Newman D.J., Cragg G.M., Snader K.M. (2000). The influence of natural products upon drug discovery. Nat. Prod. Rep..

[7] Buss A.D., Cox B., Waigh R.D., Abraham D.J. (2003). Burger’s Medicinal Chemistry and Drug Discovery.

[8] Galeotti N., Di Cesare M.L., Mazzanti G., Bartolini A., Ghelardini C. (2002). Menthol: A natural analgesic compound. Neurosci. Lett..

[9] Da Silva M.S., De Sousa D.P., Medeiros V.M., Folly M.A.B., Tavares J.F., Barbosa-Filho J.M. (2008). Alkaloid, flavonoids, and pentacyclic triterpenoids of *Maytenus obtusifolia* Mart. Biochem. Syst. Ecol..

[10] De Sousa D.P., De Almeida R.N. (2005). Neuroleptic-like properties of chloroform extract of *Maytenus obtusifolia*. Biol. Pharm. Bull..

[11] Bispo M.D., Mourão R.H.V., Franzotti E.M., Bomfim K.B.R., Arrigoni-Blank M.F., Moreno M.P.N., Marchioro M., Antoniolli A.R. (2001). Antinociceptive and antiedematogenic effects of the aqueous extract of *Hyptis pectinata* leaves in experimental animals. J. Ethnopharmacol..

[12] Menezes I.A.C., Marques M.S, Santos T.C., Dias K.S., Silva A.B., Mello I., Lisboa A.C.C.D., Alves P.B., Cavalcanti S.C.H., Marçal R.M., Antoniolli A.R. (2007). Antinociceptive effect and acute toxicity of the essential oil of *Hyptis fruticosa* in mice. Fitoterapia.

[13] Dantas M.C., Oliveira F.S., Bandeira S.M., Batista J.S., Dias J.C., Barreto P.A., Antoniolli A.R., Marchioro M. (2004). Central nervous system effects of the crude extract of *Erythrina velutina* on rodents. J. Ethnopharmacol..

[14] Craveiro A.A., Fernandes A.G., Andrade C.H.S., Matos F.J.A., Alencar J.W., Machado M.I.L. (1981). Óleos Essenciais de Plantas do Nordeste.

[15] Lis-Balchin M., Hart S. (1999). Studies on the mode of action of the essential oil of lavender (*Lavandula angustifolia* P. Miller). Phytother. Res..

[16] Pultrini A.M., Galindo L.A., Costa M. (2006). Effects of the essential oil from *Citrus aurantium* L. in experimental anxiety models in mice. Life Sci..

[17] Almeida R.N., Motta S.C., Leite J.R. (2003). Óleos essenciais com propriedades anticonvulsivantes. Bol. Latinoam. Caribe Plantas Med. Aromat..

[18] Santos F.A., Jeferson F.A., Santos C.C., Silveira E.R., Rao V.S.N. (2005). Antinociceptive effect of leaf essential oil from *Croton sonderianus* in mice. Life Sci..

[19] De Sousa D.P., Raphael E., Brocksom U., Brocksom T.J. (2004). Antinociceptive profile of 2-phenylselenenyl-1,8-cineole in mice. Biol. Pharm. Bull..

[20] De Sousa D.P., Oliveira F.S., Almeida R.N. (2006). Evaluation of the central activity of hydroxydihydrocarvone. Biol. Pharm. Bull..

[21] De Sousa D.P., Júnior E.V.M., Oliveira F.S., Almeida R.N., Nunes X.P. (2007). Synthesis and analgesic-like effect of (6*R*, 4*S*)-*p*-mentha-1,8-dien-6-yl-methylene-*p*-toluenesulfonamide. Z. Naturforsch..

[22] Oliveira F.S., De Sousa D.P., De Almeida R.N. (2008). Antinociceptive Effect of Hydroxydihydrocarvone. Biol. Pharm. Bull..

[23] De Almeida R.N., Araújo D.A.M., Gonçalves J.C.R., Montenegro F.C., De Sousa D.P., Leite J.R., Mattei R., Benedito M.A.C., Carvalho J.G.B., Cruz J.S., Maia J.G.S. (2009). Rosewood oil induces sedation and inhibits compound action potential in rodents. J. Ethnopharmacol..

[24] Peana A.T., D'Aquila P.S., Chessa M.L., Moretti M.D.L., Serra G., Pippia P. (2003). Linalool produces antinociception in two experimental models of pain. Eur. J. Pharmacol..

[25] Peana A.T., Rubattu P., Piga G.G., Fumagalli S., Boatto G., Pippia P., De Montis M.G. (2006). Involvement of adenosine A1 and A2A receptors in (-)-linalool-induced antinociception. Life Sci..

[26] Peana A.T., Marzocco S., Popolo A., Pinto A. (2006). (-)-Linalool inhibits *in vitro* NO formation: Probable involvement in the antinociceptive activity of this monoterpene compound. Life Sci..

[27] Peana A.T., De Montis M.G., Nieddu E., Spano M.T., D'Aquila P.S., Pippia P. (2004). Profile of spinal and supra-spinal antinociception of (-)-linalool. Eur. J. Pharmacol..

[28] Klein A.H., Sawyer C.M., Carstens M.I., Tsagareli M.G., Tsiklauri N., Carstens E. (2010). Topical application of l-menthol induces heat analgesia, mechanical allodynia, and a biphasic effect on cold sensitivity in rats. Behav. Brain Res..

[29] Ahmed M., Amin S., Islam M., Takahashi M., Okuyama E., Hossain C.F. (2000). Analgesic principle from *Abutilon indicum*. Pharmazie.

[30] Ohkubo T., Shibata M. (1997). The selective capsaicin antagonist capsazepine abolishes the antinociceptive action of eugenol and guaiacol. J. Dental Res..

[31] Lee M.H., Yeon K.-Y., Park C.-K., Li H.-Y., Fang Z., Kim M.S., Choi S.-Y., Lee S.J., Lee S., Park K., Lee J.-H., Kim J.S., Oh S.B. (2005). Eugenol inhibits calcium currents in dental afferent neurons. J. Dent. Res..

[32] De Sousa D.P., Júnior E.V.M., Oliveira F.S., Almeida R.N., Nunes X.P., Barbosa-Filho J.M. (2007). Antinociceptive activity of structural analogues of rotundifolone: structure-activity relationship. Z. Naturforsch..

[33] Rice K.C., Wilson R.S. (1976). 3-Isothujone, a small nonnitrogenous molecule with antinociceptive activity in mice. J. Med. Chem..

[34] Gonçalves J.C.R., Oliveira F.S., Benedito R.B., De Sousa D.P., Almeida R.N., Araújo D.A.M. (2008). Antinociceptive activity of (-)-carvone: evidence of association with decreased peripheral nerve excitability. Biol. Pharm. Bull..

[35] Wheeler-Aceto H., Porreca F., Cowan A. (1990). The rat paw formalin test: A comparison of noxious agents. Pain.

[36] Amaral J.F., Silva M.I.G., Neto M.R.A., Neto P.F.T., Moura B.A., Melo C.T.V., Araújo F.L.O., De Sousa D.P., Vasconcelos P.F., Vasconcelos S.M., Sousa F.C.F. (2007). Antinociceptive effect of the monoterpene R-(+)-limonene in mice. Biol. Pharm. Bull..

[37] Heapy C.G., Jamieson A., Russel N.J.W. (1987). Afferent C-fiber and A- delta fiber activity in models of inflammation. Br. J. Pharmacol..

[38] Murray C.W., Porreca F., Cowan A. (1988). Methodological refinements to the mouse paw formalin test. J. Pharmacol. Toxicol. Methods.

[39] Rujjanawate C., Kanjanapothi D., Panthong A. (2003). Pharmacological effect and toxicity of alkaloids from *Gelsemium elegans* Benth. J. Ethnopharmacol..

[40] Chapman C.R., Casey K.L., Dubner R., Foley K.M., Gracely R.H., Reading A.E. (1985). Painmeasurement: An overview. Pain.

[41] Grumbach L., Knighton R.S., Dumke P.R. (1966). The Prediction of Analgesic Activity in Man by Animal Testing.

[42] Parkhouse J., Pleuvry B.J. (1979). Analgesic Drug.

[43] Koster R., Anderson M., Beer E.J. (1959). Acetic acid for analgesic screening. Fed. Proc..

[44] Collier H.O.J., Dinnen L.C., Johnson C.A., Schneider C. (1968). The abdominal constriction response and its suppression by analgesic drugs in mouse. Br. J. Pharmacol..

[45] Jansen P.A.J., Niemegeers C.J.E., Dony J.G.H. (1963). The inhibitory effect of fentanyl and other morphine-like analgesics on the warm water induced tail withdrawal reflex in rats. Arzneim Forsch. Drug Res..

[46] Grotto M., Sulman F.G. (1967). Modified receptable method for animal analgesimetry. Arch. Int. Pharmacodyn..

[47] Kohlert C., van Rensen I., März R., Schindler G., Graefe E.U., Veit M. (2000). Bioavailability and Pharmacokinetics of Natural Volatile Terpenes in Animals and Humans. Planta Med..

[48] Millet Y., Jouglard J., Steinmetz M.D., Tognetti P., Joanny P., Arditti J. (1981). Toxicity of some essential plant oils. Clinical and experimental study. Clin. Toxicol..

[49] Burkhard P.R., Burkhard K., Haenggli C., Landis T. (1999). Plant-induced seizures: reappearance of an old problem. J. Neurol..

[50] Rao V.S.N., Menezes A.M.S., Viana G.S.B. (1990). Effect of myrcene on nociception in mice. J. Pharm. Pharmacol..

[51] Lorenzetti B.B., Souza G.E., Sarti S.J., Santos Filho D., Ferreira S. (1991). Myrcene mimics the peripheral analgesic activity of lemongrass tea. J. Ethnopharmacol..

[52] Orhan I., Kuepeli E., Aslan M., Kartal M., Yesilada E. (2006). Bioassay-guided evaluation of anti-inflammatory and antinociceptive activities of pistachio, *Pistacia vera* L. J. Ethnopharmacol..

[53] Him A., Ozbek H., Turel I., Oner A.C. (2008). Antinociceptive activity of alpha-pinene and fenchone. Pharmacol. Online.

[54] Santos F.A., Rao V.S.N., Silveira E.R. (1998). Investigations on the antinociceptive effect of *Psidium guajava* leaf essential oil and its major constituents. Phytother. Res..

[55] Liapi C., Anifandis G., Chinou I., Kourounakis A.P., Theodosopoulos S., Galanopoulou P. (2007). Antinociceptive properties of 1,8-cineole and β-pinene, from the essential oil of *Eucalyptus camaldulensis* leaves, in rodents. Planta Med..

[56] Amaral J.F., Silva M.I.G., Neto M.R.A., Neto P.F.T., Moura B.A., Melo C.T.V., Araújo F.L.O., De Sousa D.P., Vasconcelos P.F., Vasconcelos S.M., Sousa F.C.F. (2007). Antinociceptive effect of the monoterpene R-(+)-limonene in mice. Biol. Pharm. Bull..

[57] Santos F.A., Rao V.S. (2000). Antiinflammatory and antinociceptive effects of 1,8-cineole a terpenoid oxide present in many plant essential oils. Phytother. Res..

[58] Almeida R.N., Hiruma C.A., Barbosa-Filho J.M. (1996). Analgesic effect of rotundifolone in rodents. Fitoterapia.

[59] Sousa P.J.C., Linard C.F.B.M., Azevedo-Batista D., Oliveira A.C., Coelho-de-Souza A.N., Leal-Cardoso J.H. (2009). Antinociceptive effects of the essential oil of *Mentha* x* villosa* leaf and its major constituent piperitenone oxide in mice. Braz. J. Med. Biol. Res..

[60] Yamahara J., Matsuda H., Watanabe H., Sawada T., Fujimura H. (1980). Biologically active principles of crude drugs. Analgesic and anti-inflammatory effects of "Keigai (*Shizonepeta tenuifolia* Briq)". Yakugaku Zasshi.

[61] Melo M.S., Sena L.C.S., Barreto F.J.N., Bonjardim L.R., Almeida J.R.G.S., Lima J.T., De Sousa D.P., Quintans-Junior L.J. (2010). Antinociceptive effect of citronellal in mice. Pharm. Biol..

[62] Quintans-Junior L.J., Melo M.S., De Sousa D.P., Araujo A.A.S., Onofre A.C.S., Gelain D.P., Gonçalves J.C.R., Araujo D.A.M., Almeida J.R.G.S., Bonjardim L.R. (2010). Antinociceptive effects of citronellal in formalin-, capsaicin-, and glutamate-induced orofacial nociception in rodents and its action on nerve excitability. J. Orofac. Pain.

[63] Ortiz M.I., Ramírez-Montiel M.L., González-García M.P., Ponce-Monter H.A., Castañeda-Hernández G., Cariño-Cortés R. (2010). The combination of naproxen and citral reduces nociception andgastric damage in rats. Arch. Pharm. Res..

[64] Batista P.A., Werner M.F.P., Oliveira E.C., Burgos L., Pereira P., Brum L.F.S., Santos A.R.S. (2008). Evidence for the involvement of ionotropic glutamatergic receptors on the antinociceptive effect of (-)-linalool in mice. Neurosci. Lett..

[65] Batista P.A., Werner M.F.P., Oliveira E.C., Burgos L., Pereira P., Brum L.F.S., Story G.M., Santos A.R.S. (2010). The Antinociceptive Effect of (-)-Linalool in Models of Chronic Inflammatory and Neuropathic Hypersensitivity in Mice. J. Pain.

[66] Peana A.T., De Montis M.G., Sechi S., Sircana G., D'Aquila P.S., Pippia P. (2004). Effects of (-)-linalool in the acute hyperalgesia induced by carrageenan, L-glutamate and prostaglandin E_2_. Eur. J. Pharmacol..

[67] Wang T., Zhang R., Chen C., Wu H., Wu Q., Wang N., Mi S. (2009). Comparison on analgesic effect of (+)-bornyl monomaleate and natural borneol. Zhongyao Yaoli Yu Linchuang.

[68] Angeles-Lopez G., Perez-Vasquez A., Hernandez-Luis F., Deciga-Campos M., Bye R., Linares E., Mata R. (2010). Antinociceptive effect of extracts and compounds from *Hofmeisteria schaffneri*. J. Ethnopharmacol..

[69] Guimaraes A.G., Oliveira G.F., Melo M.S., Cavalcanti S.C.H., Antoniolli A.R., Boniardim L.R., Silva F.A., Santos J.P.A., Rocha R.F., Moreira J.C.F., Araujo A.A.S., Gelain D.P., Quintans L.J. (2010). Bioassay-guided evaluation of antioxidant and antinociceptive activities of carvacrol. Basic Clin. Pharmacol. Toxicol..

[70] Abdel-Fattah A.M., Matsumoto K., Watanabe H. (2000). Antinociceptive effects of *Nigella sativa* oil and its major component, thymoquinone, in mice. Eur. J. Pharmacol..

[71] Sulaiman M.R., Perimal E.K., Zakaria Z.A., Mokhtar F., Akhtar M.N., Lajis N.H., Israf D.A. (2009). Preliminary analysis of the antinociceptive activity of zerumbone. Fitoterapia.

[72] Chavan M.J., Wakte P.S., Shinde D.B. (2010). Analgesic and anti-inflammatory activity of Caryophyllene oxide from *Annona squamosa* L. bark. Phytomedicine.

[73] Okugawa H., Ueda R., Matsumoto K., Kawanishi K., Kato K. (2000). Effects of sesquiterpenoids from "Oriental incenses" on acetic acid-induced writhing and D2 and 5-HT2A receptors in rat brain. Phytomedicine.

[74] Kurian R., Arulmozhi D.K., Veeranjaneyulu A., Bodhankar S.L. (2006). Effect of eugenol on animal models of nociception. Indian J. Pharmacol..

[75] Daniel A.N., Sartoretto S.M., Schmidt G., Caparroz-Assef S.M., Bersani-Amado C.A., Cuman R.K.N. (2009). Anti-inflammatory and antinociceptive activities of eugenol essential oil in experimental animal models. Rev. Bras. Farmacogn..

[76] Peana A.T., Chessa G., Carta G., Delogu G., Fabbri D. (2004). Eugenol, bis- eugenol and synthesized related-dimer compounds produce antinociception in the acetic acid-induced-writhing responses. Curr. Top. Phytochem..

[77] Yano S., Suzuki Y., Yuzurihara M., Kase Y., Takeda S., Watanabe S., Aburada M., Miyamoto K. (2006). Antinociceptive effect of methyleugenol on formalin-induced hyperalgesia in mice. Eur. J. Pharmacol..

[78] De Lima A.B., Santana M.B., Cardoso A.S., da Silva J.K.R., Maia J.G.S., Carvalho J.C.T., Sousa P.J.C. (2009). Antinociceptive activity of 1-nitro-2-phenylethane, the main component of *Aniba canelilla* essential oil. Phytomedicine.

[79] Park S.H., Sim Y.B., Choi S.M., Seo Y.J., Kwon M.S., Lee J.K., Suh H.W. (2009). Antinociceptive profiles and mechanisms of orally administered vanillin in the mice. Arch. Pharm. Res..

[80] Junrong D., Yan Y., Ya K., Chenyuen W., Li Z., Ming Q.Z. (2007). Ligustilide attenuates pain behavior induced by acetic acid or formalin. J. Ethnopharmacol..

[81] Johnson A.J., Kumar R.A., Rasheed S.A., Chandrika S.P., Chandrasekhar A., Baby S., Subramoniam A. (2010). Antipyretic, analgesic, anti-inflammatory and antioxidant activities of two major chromenes from *Melicope lunu-ankenda*. J. Ethnopharmacol..

[82] Azaz A.D., Irtem H.A., Kurkcuoglu M., Baser K.H.C. (2004). Composition and the *in vitro* Antimicrobial Activities of the Essential Oils of some *Thymus* species. Z. Naturforsch..

[83] Mikaili P., Nezhady M.A.M., Shayegh J., Asghari M.H. (2010). Study of antinociceptive of *Thymus vulgaris* and *Foeniculum vulgare* essential oil in mouse. Int. J. Acad. Res..

